# The dynamin GTPase mediates regenerative axonal fusion in *Caenorhabditis elegans* by regulating fusogen levels

**DOI:** 10.1093/pnasnexus/pgad114

**Published:** 2023-05-09

**Authors:** Tarika Vijayaraghavan, Samiksha Dhananjay, Xue Yan Ho, Rosina Giordano-Santini, Massimo Hilliard, Brent Neumann

**Affiliations:** Neuroscience Programme, Biomedicine Discovery Institute and Department of Anatomy and Developmental Biology, Monash University, Melbourne, VIC 3800, Australia; Neuroscience Programme, Biomedicine Discovery Institute and Department of Anatomy and Developmental Biology, Monash University, Melbourne, VIC 3800, Australia; Clem Jones Centre for Ageing Dementia Research, Queensland Brain Institute, The University of Queensland, Brisbane, QLD 4072, Australia; Clem Jones Centre for Ageing Dementia Research, Queensland Brain Institute, The University of Queensland, Brisbane, QLD 4072, Australia; Clem Jones Centre for Ageing Dementia Research, Queensland Brain Institute, The University of Queensland, Brisbane, QLD 4072, Australia; Neuroscience Programme, Biomedicine Discovery Institute and Department of Anatomy and Developmental Biology, Monash University, Melbourne, VIC 3800, Australia

**Keywords:** axonal fusion, dynamin GTPases, DYN-1, EFF-1, axon regeneration

## Abstract

Axonal fusion is a neuronal repair mechanism that results in the reconnection of severed axon fragments, leading to the restoration of cytoplasmic continuity and neuronal function. While synaptic vesicle recycling has been linked to axonal regeneration, its role in axonal fusion remains unknown. Dynamin proteins are large GTPases that hydrolyze lipid-binding membranes to carry out clathrin-mediated synaptic vesicle recycling. Here, we show that the *Caenorhabditis elegans* dynamin protein DYN-1 is a key component of the axonal fusion machinery. Animals carrying a temperature-sensitive allele of *dyn-1(ky51)* displayed wild-type levels of axonal fusion at the permissive temperature (15°C) but presented strongly reduced levels at the restrictive temperature (25°C). Furthermore, the average length of regrowth was significantly diminished in *dyn-1(ky51)* animals at the restrictive temperature. The expression of wild-type DYN-1 cell-autonomously into *dyn-1(ky51)* mutant animals rescued both the axonal fusion and regrowth defects. Furthermore, DYN-1 was not required prior to axonal injury, suggesting that it functions specifically after injury to control axonal fusion. Finally, using epistatic analyses and superresolution imaging, we demonstrate that DYN-1 regulates the levels of the fusogen protein EFF-1 post-injury to mediate axonal fusion. Together, these results establish DYN-1 as a novel regulator of axonal fusion.

**Significance Statement**

A severed nerve can restore its original functionality by reestablishing the connection between its separated axon fragments in a process known as axonal fusion. Using the nematode worm *Caenorhabditis elegans*, we and others have revealed a key role for the apoptotic machinery in the recognition of the two separated fragments and for the fusogen EFF-1 in merging the two membranes. Dynamin GTPases are evolutionarily conserved proteins involved in a variety of cellular processes including apoptosis and synaptic vesicle recycling. In this study, we establish a role for the *C. elegans* dynamin GTPases (DYN-1) in regenerative axonal fusion. We further report that DYN-1 is important for the expression of the fusogen protein EFF-1 to promote axonal fusion and repair.

## Introduction

Regenerative axonal fusion promotes the functional recovery of injured neurons by reuniting severed segments of an axon (1–5). This process has been best characterized in the *Caenorhabditis elegans* (*C. elegans*) posterior lateral microtubule (PLM) axons, which undergo spontaneous regrowth, reconnection, and fusion that restores the structure and function of the neuron after injury (2–5). Molecules previously implicated in the recognition of apoptotic cells have been shown to be repurposed in the nerve repair pathway to promote regrowth and reconnection after axonal injury. For example, immediately following cell death, the normally internalized phospholipid phosphatidylserine (PS) is exposed on the outer leaflet of the plasma membrane where it is bound by the PS receptor PSR-1 to mediate cell corpse recognition (6). Meanwhile, the ABC transporter protein CED-7 and transthyretin protein TTR-52 facilitate the release of PS-exposing vesicles from the dying cell to the extracellular space to further attract engulfing cells (7). Simultaneously, the intracellular adaptor CED-1/LRP1 binds to TTR-52 and the lipid-binding protein NRF-5 at the plasma membrane and initiates a signaling cascade via the CED-1 adaptor protein CED-6/GULP (7–9). During the axonal fusion process, all of these proteins except CED-1 function to enable regrowth and recognition between severed axon fragments (3). Although the GTPase protein DYN-1/dynamin also functions in the recognition of dying cells (10), its role in axonal repair has not previously been reported.

The dynamin GTPases are large proteins that mediate vesicle scission during endocytosis and synaptic vesicle recycling. Pivotal studies conducted in *Drosophila melanogaster* reported the participation of Shibire/dynamin 1 in neurotransmission and synaptic vesicle recycling (11–14). Other proteins linked to neurotransmission have previously been reported to participate in the regeneration of the *C. elegans* PLM axons, including the microtubule-associated motor protein UNC-57/endophilin (15). Since the mammalian dynamin proteins are known to associate with microtubules (16) and function in the apoptotic recognition pathway (10, 17), we hypothesized that the dynamin GTPases may also have a role during regenerative axonal fusion. Here, we confirm this hypothesis by establishing that the DYN-1 protein is important for facilitating axonal fusion and regrowth after injury.

In *C. elegans*, axonal fusion is reliant on the transmembrane glycoprotein epithelial fusion failure-1 (EFF-1), which is structurally homologous to class II viral fusion proteins (18, 19). Animals lacking EFF-1 display extensive branching and physical connection between severed axon segments but fail to fuse these segments (3, 20). Further investigations using the *C. elegans* bilateral pair of PLM neurons revealed that EFF-1 mediates homotypic fusion between PLM axon segments in a manner resembling that in hypodermal and muscle cells (3, 20–24). In addition, the metalloprotease ADM-4 stabilizes EFF-1 to allow the membranes to merge (25). A recent study also reported that the small GTPase RAB-5 facilitates the endosomal recycling of EFF-1 in the PLM soma and increases the capacity of the neuron to undergo regenerative axonal fusion (26). Other interactors of EFF-1 have been identified in the context of cell–cell fusion, epidermal wound healing, and neuronal maintenance (24, 27–30). However, how EFF-1 is regulated in the axon where it is required to drive the fusion of separated axon segments has not previously been reported. Here, we demonstrate that DYN-1 controls the levels of EFF-1 to promote axonal fusion.

## Results

### DYN-1 is important for axonal fusion

**Fig. 1.**
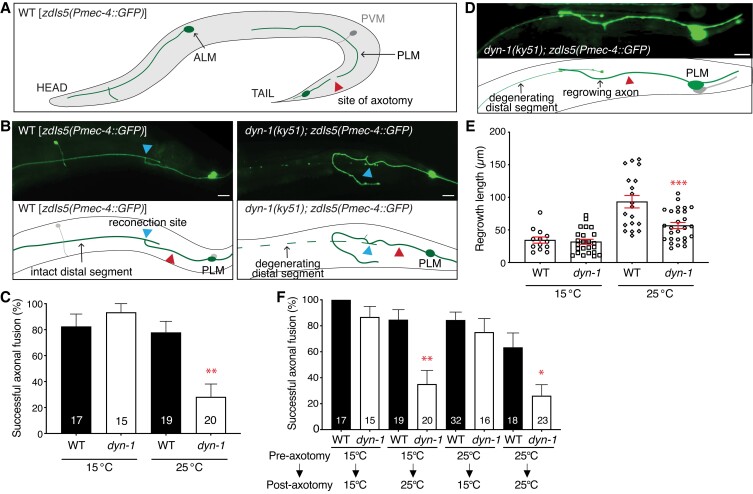
DYN-1 is important for axonal fusion. A) Illustration of the mechanosensory neurons in *C. elegans* after axotomy. PLM, posterior lateral microtubule neuron; ALM, anterior lateral microtubule neuron; PVM, posterior ventral microtubule neuron. B) The PLM neuron was cut ∼50 µm from the cell body (represented by a red triangle in the schematics) and successfully fused in the control animal carrying the *zdIs5(Pmec-4::GFP)* transgene (left image and scheme) but unsuccessfully in the *dyn-1(ky51)* animal (right image and scheme). The site of reconnection is represented by a blue triangle. A successful event was recorded when reconnected fragments maintained the integrity of the axon 48 h of injury (left), and an unsuccessful event was when the distal fragment displayed degeneration after reconnection (right). Scale bars represent 10 µm. C) Quantification of the axonal fusion levels in WT and *dyn-1* animals 48 h after UV laser axotomy of the PLM axon at permissive (15°C) and restrictive temperature (25°C). Error bars represent standard error of proportion; *P*-values calculated using Fisher's exact tests; **P < 0.01. Numbers within individual bars indicate the number of reconnection events per genotype, where *n* ≥ 15 reconnection events. D) Example of a regrowth event, whereby the length of regrowth is calculated from the site of injury (red triangle in the schematic) to the distal most point of the growing fragment, 24 h post-axotomy. E) Analysis of regrowth 24 h after UV laser transection in WT and *dyn-1* animals at permissive and restrictive temperatures. *dyn-1* animals display a significant defect at 25°C but not at 15°C, compared with WT animals. *P*-values calculated using the unpaired *t* test where ***P < 0.001. F) Axonal fusion levels in WT and *dyn-1* animals were grown at 15°C before and after axotomy (bars 1 and 2), at 15°C before axotomy and at 25°C afterward (bars 3 and 4), at 25°C before axotomy and at 15°C after axotomy (bars 5 and 6), and at 25°C before axotomy followed by 25°C post-injury (bars 7 and 8). Error bars represent standard error of proportion; *P*-values calculated using Fisher's exact tests; *P < 0.05 and **P < 0.005. Numbers within individual bars indicate the number of reconnection events per genotype, where *n* ≥ 15 reconnection events.

To analyze the role of DYN-1 in axonal fusion, we used the only viable mutant allele (*ky51*), which introduces a P70S point mutation that results in a temperature-sensitive loss of DYN-1 function: the DYN-1 protein degrades at restrictive temperatures (25°C) but functions normally at permissive temperatures (15°C) (31, 32). The P70S allele affects the GTPase domain, which is required to hydrolyze the lipid membrane and facilitate the oligomerization of DYN-1 proteins at the site of scission (31, 32). To study regenerative axonal fusion, the *C. elegans* PLM axon was severed ∼50 µm from the cell body with a Micropoint UV laser system (Fig. 1A). Successful fusion events were recorded 48 h after injury only when membrane integrity and cytoplasmic continuity were established between the two fragments (Fig. 1B) (3–5). At the restrictive temperature, *dyn-1(ky51)* animals displayed a strong reduction in the level of axonal fusion (Fig. 1C). However, when grown at 15°C (permissive for DYN-1 function), the level of fusion in *dyn-1(ky51)* animals was indistinguishable from wild-type (WT) controls (Fig. 1C). We further analyzed regeneration by recording the length of regrowth in axons that did not establish physical contact with their severed segments (Fig. 1D). The length of axonal regrowth 24 h after ablation was significantly reduced in *dyn-1* animals grown at the restrictive temperature (Fig. 1E). Thus, DYN-1 is involved in both axonal fusion and regrowth pathways.

To understand the temporal regulation of DYN-1 during axonal fusion, we grew *dyn-1(ky51)* animals at either 15°C (permissive temperature) or 25°C (restrictive temperature) prior to axotomy before either keeping them at the same conditions or shifting them to the alternative temperature after axotomy. As shown in Fig. 1F, *dyn-1(ky51)* animals displayed significantly reduced axonal fusion when incubated at 15°C pre-axotomy and transferred to 25°C immediately afterward. In contrast, animals incubated at 25°C pre-axotomy and transferred at 15°C post-axotomy displayed no defect in axonal fusion (Fig. 1F). Thus, these experiments suggest that DYN-1 is important after injury for axonal fusion. Furthermore, additional temperature-shift experiments indicate that DYN-1 functions throughout the regenerative process to promote axonal fusion (Fig. S1A and B).

### DYN-1 likely functions cell-autonomously to facilitate regrowth and repair

**Fig. 2.**
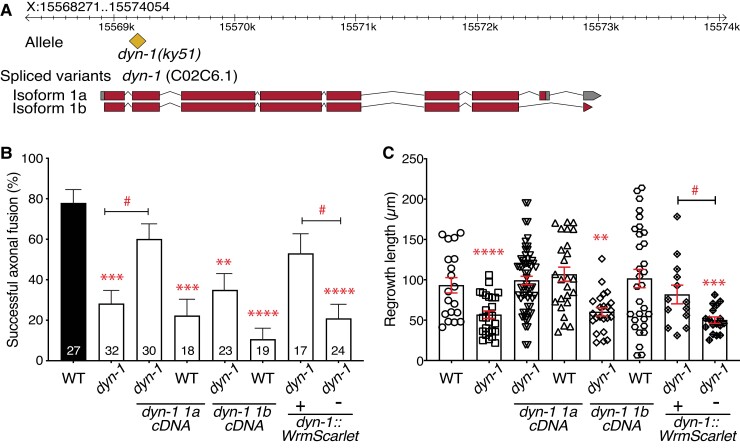
DYN-1 isoform 1a functions cell-autonomously to mediate axonal regeneration. A) Representative chart showing the position of the *dyn-1(ky51)* allele and the spliced variants used in this study. The yellow diamond icon represents the location of the point mutation, and the solid red rectangles represent the exons of the *dyn-1* transcript, introns as black lines, and untranslated regions in gray. B) Quantification of successful axonal fusion events in PLM-specific *dyn-1(ky51)* knock-in lines *sybIs2534(Pmec-17::dyn-1 1a cDNA::rab-3 UTR)* and *sybIs2535(Pmec-17::dyn-1 1b cDNA::rab-3 UTR)* and in animals with WT dynamin expressed from its endogenous promoter (*Pdyn-1::dyn-1::WrmScarlet* (5 ng/µL)) compared with controls. Animals were incubated at 25°C post-axotomy. Error bars represent standard error of proportion; *P*-values calculated using Fisher's exact tests; *P < 0.05, **P < 0.005, ***P < 0.001, and ****P < 0.0001. Numbers within individual bars indicate the number of reconnection events per genotype, where *n* ≥ 15 reconnection events. C) Quantification of regrowth lengths in WT and *dyn-1(ky51)* animals, together with the transgenic rescue lines. Animals were incubated at 25°C post-axotomy. *P*-values calculated using Student’s *t* tests (in group comparisons designated with asterisks; between group comparisons designated with hash symbols); *P < 0.05, **P < 0.005, ***P < 0.001, and ****P < 0.0001, *n* ≥ 30 animals per genotype.

To confirm the involvement of DYN-1 in axonal regrowth and fusion, we performed genetic rescue experiments by expressing WT *dyn-1* specifically in the six mechanosensory neurons (including the two PLM neurons) of *dyn-1(ky51)* animals. Transgenic strains harboring extrachromosomal arrays of WT *dyn-1* displayed partial rescue of the deficiencies in axonal fusion and regrowth lengths (Fig. S1C). To further confirm the role of DYN-1 in these phenotypes, we used CRISPR-Cas9 to introduce either of the two dynamin isoforms (1a and 1b) into *dyn-1(ky51)* animals (Fig. 2A). Isoform 1a partially, but significantly, rescued the fusion defect and fully rescued the regrowth length defect (Fig. 2B). Interestingly, isoform 1b failed to rescue either defect, suggesting that only isoform 1a is involved in axonal repair (Fig. 2B and C; bars 3–6). This rescue data demonstrate that DYN-1 likely functions cell-autonomously to mediate axonal fusion and regrowth. We next expressed *dyn-1* from its endogenous promoter in *dyn-1(ky51)* animals to determine whether this could further enhance the levels of axonal fusion and the length of regrowth. Interestingly, this transgene only partially rescued the axonal fusion and fully rescued the regrowth length defects in *dyn-1* animals to similar levels as those seen with the cell-autonomous rescue transgenes (Fig. 2B and C; bars 7 and 8). Together, these data suggest that DYN-1 may act cell-autonomously in the PLM neuron to promote axonal repair.

### DYN-1 interacts with EFF-1 to promote axonal fusion

**Fig. 3.**
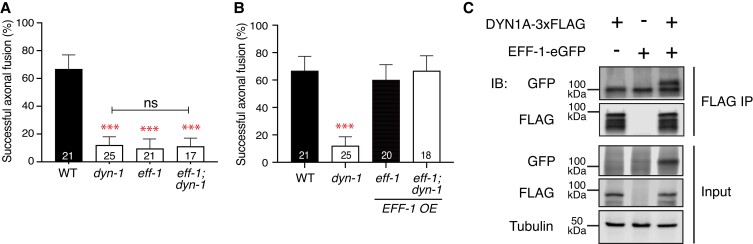
DYN-1 interacts with EFF-1 in the context of axonal fusion. Graphs representing axonal fusion levels of A) single *dyn-1(ky51)* and *eff-1(ok1021)* mutants and double *eff-1(ok1021); dyn-1(ky51)* mutants and B) compared with animal with EFF-1 overexpression (OE) [strain QH4748 (*vdEx662[Pmec-4::EFF-1::GFP); Pmec-4::mCherry])*] in single and double mutants. Error bars represent standard error of proportion; numbers within individual bars indicate the number of reconnection events per genotype, where *n* ≥ 15 reconnection events. C) DYN-1A interacts with EFF-1 *in vitro*. HEK293T cells were transfected with plasmids encoding DYN-1-3xFLAG and/or EFF-1A-eGFP. Complex formation was detected by immunoprecipitation (IP) with an anti-FLAG antibody, followed by immunoblotting (IB) with an anti-GFP antibody. Blot of one experiment. Uncropped images are shown in Fig. S3.

The nematode-specific fusogen EFF-1 is a key molecule for the process of axonal fusion (3) and is known to interact with DYN-1 to regulate hypodermal cell fusion (29). Hence, we examined the genetic interaction between *eff-1* and *dyn-1* in the context of axonal fusion. Axonal fusion levels were assessed in *eff-1(ok1021)* and *dyn-1(ky51)* single- and double-mutant animals at the restrictive temperature (25°C). Both *dyn-1* and *eff-1* single mutants displayed strong reductions in fusion levels compared with the wild type (Fig. 3A). Importantly, *eff-1; dyn-1* double-mutant animals also displayed significant reductions in fusion levels that were not worse than either of the single-mutant strains (Fig. 3A), indicating that *dyn-1* and *eff-1* function in the same genetic pathway. Loss of EFF-1 did not affect the average length of regrowth and did not worsen the level observed in *dyn-1(ky51)* mutants (Fig. S2A). Thus, *dyn-1* and *eff-1* appear to function in the same genetic pathway during axonal fusion, with EFF-1 not required for axonal regrowth.

To further examine the interaction between EFF-1 and DYN-1, we overexpressed EFF-1 in animals carrying the *dyn-1(ky51)* mutation. To overexpress EFF-1, we used the *vdEx662* transgene, which contains an mCherry fluorophore driven by a PLM-specific promoter (*mec-4*) and a GFP-tagged version of EFF-1 expressed under the same promoter (3). After injury, EFF-1 localizes to the membrane to fuse the separated segments back together (3) (Fig. S2B–D). We performed UV laser axotomy in EFF-1 overexpressing animals carrying the *dyn-1(ky51)* mutation and observed WT levels of fusion, suggesting that EFF-1 lies downstream to DYN-1 (Fig. 3B).

**Fig. 4.**
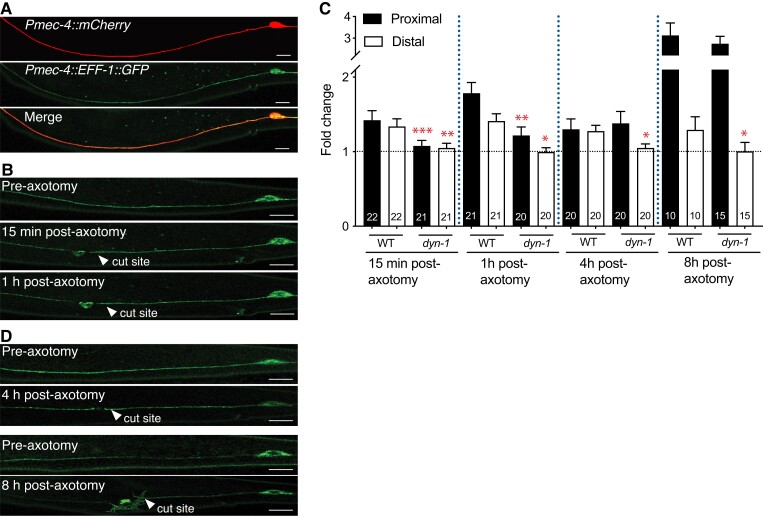
DYN-1 is important for the expression of EFF-1 immediately after injury. A) The QH4748 strain was introduced into *dyn-1(ky51)* animals, and expression levels were assessed before and after axotomy. *Pmec-4::mCherry* (top) is a reference channel, and *Pmec-4::EFF-1::GFP* (middle) overexpresses EFF-1 in the PLM axon. B) Pre- and postaxotomy images taken in WT and *dyn-1* animals. L4 worms were incubated for a minimum of 2 h at 25°C before preaxotomy images were taken and transferred to 25°C after axotomy. C, D) Animals were imaged at 15 min, 60 min, 4 h, and 8 h postaxotomy. Fold change calculated relative to preaxotomy images.

To examine if the DYN-1 protein forms a complex with EFF-1, we performed coimmunoprecipitation experiments. Full-length DYN-1A tagged with FLAG (DYN-1-3xFLAG) and EFF-1A tagged with GFP (EFF-1A-eGFP) were expressed in human embryonic kidney (HEK) 293T cells, and DYN-1A was immunoprecipitated with anti-FLAG antibodies. As shown in Figs. 3C and S3, EFF-1 coprecipitated with DYN-1A, suggesting that these two proteins can physically interact.

### DYN-1 is important for the expression of EFF-1 immediately after injury

Using superresolution microscopy and a UV laser system, we measured the levels of EFF-1 at different time points after injury. Interestingly, we found that EFF-1 levels were significantly reduced in *dyn-1(ky51)* animals on both proximal and distal axon segments at the earliest time points following injury (measured at 15 min and 1 h postaxotomy; Fig. 4A–C). At later time points post-axotomy (4- and 8-h), EFF-1 levels were significantly reduced only in the distal segment, with the proximal segment retaining similar levels to the wild type (Fig. 4C and D). EFF-1 expression levels were unchanged between *dyn-1(ky51)* and WT animals 24 h postaxotomy, and the loss of *dyn-1* did not affect the localization of EFF-1 to the membrane post-axotomy (Fig. S2). Overall, these data imply that DYN-1 is essential for the correct regulation of EFF-1 levels soon after injury, but not so at later time points in the proximal segment.

**Fig. 5.**
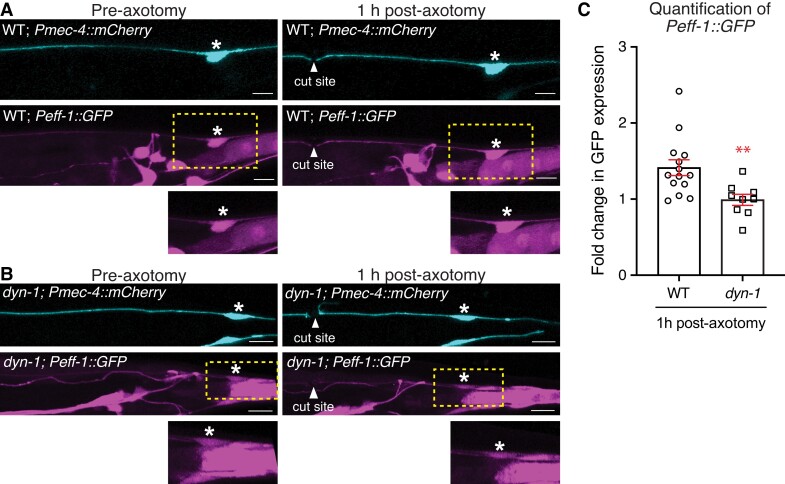
DYN-1 is important for EFF-1 transcriptional activity. A) Imaging of the PLM neurons before axotomy (left panels) and 1 h post-axotomy (right panels) in WT animals expressing mCherry from the *mec-4* promoter (*Pmec-4::mCherry*, top panels) and GFP from the *eff-1* promoter (*Peff-1::GFP*, middle and bottom panels). Asterisks highlight the position of the PLM cell body; arrowheads show the site of axotomy; yellow, dashed boxes show the approximate region enlarged in the bottom panels; scale bars represent 10 µm. B) Imaging of the PLM neurons before axotomy (left panels) and 1 h post-axotomy (right panels) in *dyn-1(ky51)* animals expressing mCherry from the *mec-4* promoter (*Pmec-4::mCherry*, top panels) and GFP from the *eff-1* promoter (*Peff-1::GFP*, middle and bottom panels). Asterisks highlight the position of the PLM cell body; arrowheads show the site of axotomy; yellow, dashed boxes show the approximate region enlarged in the bottom panels; scale bars represent 10 µm. C) Quantification of GFP expressed from the *eff-1* promoter in the cell body in WT and *dyn-1(ky51)* animals 1 h post-axotomy. Statistical analyses between WT and *dyn-1* were conducted using unpaired Student’s *t* tests; **P < 0.005, symbols represent individual data points, where *n* ≥ 9 animals per genotype per time point.

To address whether DYN-1 can affect the expression of *eff-1*, we used a *Peff-1::GFP* transgene and confocal microscopy to visualize the levels of GFP expressed from the *eff-1* promoter. We quantified the corrected total cell fluorescence (CTCF) intensity of GFP in the PLM cell body prior to axotomy and 1 h after severing the PLM axon. While WT animals displayed an increase in expression from the *eff-1* promoter, those lacking DYN-1 displayed a slight reduction in expression compared with the levels before axotomy levels (Fig. 5A–C). Thus, DYN-1 is important for the correct expression and levels of EFF-1 after axotomy.

Together, these data suggest that DYN-1 mediates axonal fusion by regulating the levels of EFF-1, which is required to fuse the two membranes once reconnection between the proximal and distal segments occurs.

## Discussion

### Characterizing DYN-1 in axonal repair

In this study, we have elucidated a novel role for the DYN-1/dynamin GTPase in axonal fusion. Axonal fusion levels in animals carrying the *dyn-1(ky51)* allele were higher at the permissive temperature and significantly lower at restrictive temperatures. Importantly, this defect was observed only after axotomy, suggesting that DYN-1 is not necessarily required for the development of the neuron but for its maintenance. This is in line with previously published reports in mice where individual gene knockouts of dynamin isoforms did not affect the development of a large nerve terminal (33). We further report that the DYN-1 protein is important for several hours post-injury to maintain regenerative axonal fusion. Our previous analysis of microtubule dynamics before and after axotomy provides further support for this notion (34). In the absence of axonal injury, *dyn-1* mutants displayed no noticeable defect in newly forming microtubules or in the polarity of microtubule growth (34). Similar observations were made by Noda et al. (35) in intact mammalian neurons, who suggested that dynamin participates in vesicular trafficking rather than microtubule sliding as a motor protein. However, our post-axotomy data suggest that DYN-1 might be involved in microtubule production, bundling, and polarity soon after injury (34). Thus, DYN-1 might regulate axonal regrowth and fusion by controlling microtubule dynamics and maintaining the polarity of the microtubule network. Indeed, microtubule regulation has previously been implicated in PLM axon regeneration wherein loss of function of microtubule-binding proteins has affected axonal repair after injury (15, 36, 37).

We propose that *dyn-1* might temporally regulate axonal regrowth and fusion depending on its availability in the cell. Both axonal fusion and regrowth lengths were rescued when WT *dyn-1* was reintroduced cell-autonomously. However, the overexpression of DYN-1 induced only axonal fusion defects, with regrowth length remaining unaffected. In a study conducted in Shibire/dynamin temperature-sensitive mutants in *D. melanogaster*, overexpression of dynamin resulted in poor neurotransmission, vacuolated mitochondria, and cross-linked microtubules (38). Since dynamin GTPases largely depend on the availability of GTP in the cell, it is likely that the imbalance of enzyme and substrate causes a delay in endosomal trafficking, thereby phenocopying the loss of gene function. It is also possible that the overexpression of dynamin in the neuron favors apoptosis or the “eat-me” signaling pathway as seen in metastatic cancer cells (39). These results may also suggest that regrowth and axonal fusion are regulated by two separate pathways, supporting previously published findings (4).

### Dynamin and EFF-1

This study sheds light on the interaction between DYN-1 and EFF-1 and the possibility that DYN-1 functions in axonal repair through two distinct pathways. EFF-1 and DYN-1 were previously implicated in hypodermal cell–cell fusion wherein DYN-1 was required for the uptake of EFF-1 from the plasma membrane to allow its recycling (29). In our data set, we observed a similar trend in EFF-1 expression in the distal fragments of axons that failed to reconnect. The expression of EFF-1 in the proximal fragment in *dyn-1* mutant animals displayed the largest difference to control axons soon after axotomy. This disruption in the accumulation of EFF-1 proximal to injury might be a direct consequence of disoriented microtubules post-injury (34), suggesting that DYN-1 may influence the transport of growth-promoting molecules by bundling microtubules. As the overexpression of EFF-1 resulted in a complete rescue of axonal fusion defects in *dyn-1* mutant animals, it suggests that EFF-1 lies downstream to DYN-1 in the fusion pathway.

Thus, there are at least three possibilities arising from our experimental data. First, DYN-1 is required in the proximal fragment transiently after injury to transport vesicles. Second, DYN-1 recycles EFF-1 vesicles in the distal fragment over the first several hours after injury. Third, DYN-1 influences the availability of functional EFF-1 in the cell body after axonal injury. The RAB-5 GTPase has previously been implicated in recycling EFF-1 in *C. elegans* embryos and in the PLM axon (29, 31). Thus, in the absence of DYN-1, the RAB-5/Rab5 GTPase likely transports and recycles EFF-1. While DYN-1 is not involved in this uptake of EFF-1 in intact PLM axons (31), it is not known whether DYN-1 is required for the endocytosis or transport of EFF-1 after injury. DYN-1 may also be required for the endocytosis of molecules in the apoptotic recognition machinery that are known to be repurposed to facilitate axonal fusion (3). Together, our data indicate that DYN-1 is a major player in axonal repair and likely elicits injury responses through multiple pathways.

## Materials and methods

### *C. elegans* maintenance

Animals were maintained on nematode growth media (NGM) at 15°C unless otherwise specified, using standard methods (40). Some strains were obtained from the *Caenorhabditis* Genetics Center (CGC), Minnesota, USA. The *dyn-1(ky51); zdIs5* strain was generated using standard genetic crossing. The CRISPR-generated strains *sybIs2534* and *sybIs2535* were designed by us and generated by SunyBiotech (Fujian Province, China). Varied concentrations (1 ng/µL, 5 ng/µL, and 10 ng/µL) of PLM-specific dynamin rescue and 5 ng/µL of dynamin promoter expressing genomic DNA with a C-terminal fluorescent tag (WrmScarlet) were generated using standard techniques (41). All double mutants were generated using standard genetic crossing techniques. A full list of strains used in this study is shown in Table S1.

### UV laser axotomy

PLM axons were severed with a Micropoint UV laser system (Andor—Oxford Instruments, Belfast, UK) attached to Zeiss Axio Imager.A2 compound microscope (Zeiss Group, Oberkochen, Germany), ∼50 µm from the cell body (2, 3). The levels of reconnection and fusion were recorded 24 and 48 h after axotomy, respectively, using a Zeiss Axio Imager.M2 microscope (Zeiss Group, Oberkochen, Germany). The length of regrowth from the proximal fragment was measured at the 24-h time point from the cut site to the distal most point. For animals that were incubated at 25°C before axotomy, gravid adults were bleached and washed to obtain synchronized eggs and incubated at 15°C for 48 h until they were viable L2 stage worms. Midlate L4 animals were obtained when L2 animals were incubated at 25°C for 16 h.

### Axotomy for *eff-1* and controls at restrictive temperatures

To circumvent the issue of high *eff-1(ok1021)* worm lethality at higher temperatures, at least 40 animals were analyzed per genotype and divided into two batches for imaging. Batch 1 was only imaged at the 24-h time point, and batch 2 was only imaged at the 48-h time point (Fig. 3).

Coimmunoprecipitation was performed as previously described (25). Briefly, HEK293T cells cultured in Dulbecco's modified Eagle's medium containing 10% fetal bovine serum were transfected with pMAX::dyn-1a::3xFLAG (pXH106) and/or pMAX::eff-1A::eGFP (pXH64) using X-tremeGENE 9 DNA Transfection Reagent (Sigma-Aldrich). Twenty-four hours post-transfection, cells were lysed with ice-cold radioimmunoprecipitation assay (RIPA) buffer [1% Triton X-100, 0.5% sodium deoxycholate, 100 mm NaCl, 0.1% SDS, 2 mm EDTA, 2 mm EGTA, 50 mm NaF, and 10 mm sodium pyrophosphate in *tris*-buffered saline (TBS)], containing complete EDTA-free protease inhibitor (Sigma-Aldrich). Following centrifugation (14,000 rpm for 30 min at 4°C), cell lysates were precleared for 1 h at 4°C with Pierce Control Agarose Resin (Thermo Fisher) and subsequently incubated with anti-FLAG M2 agarose beads (Sigma-Aldrich) for 3–4 h at 4°C. Following four, 15-min washers with ice-cold RIPA buffer, beads were eluted in 2× SDS sample buffer containing β-mercaptoethanol and dithiothreitol. SDS–polyacrylamide gel electrophoresis (PAGE) and western blot analysis were used to resolve the immunoprecipitated proteins.

Precast 4–15% acrylamide gels (Bio-Rad) were used for SDS-PAGE, and proteins were transferred to polyvinylidene difluoride (PVDF) membranes (100 V for 2 h). Membranes were blocked using TBS with 5% BSA and 0.1% Tween 20. They were then washed and incubated overnight at 4°C with primary antibodies: anti-eGFP (1:1,000) [Roche; from mouse immunoglobulin G1κ (clones 7.1 and 13.1) #11814460001] or anti-FLAG (1:1,000) (Sigma F1804). After washing, membranes were subsequently incubated for 1 h at room temperature with horseradish peroxidase-conjugated secondary antibodies (1:10,000). They were then washed extensively using TBS-T, prior to be analyzed with enhanced chemiluminescence methods and images being captured on an Odyssey Fc imaging system (LI-COR) with Image Studio Lite (LI-COR) software.

### Localization of EFF-1::GFP

To examine EFF-1 expression levels in regrowing axons, animals were first incubated at restrictive temperature for at least 2 h prior to imaging. Laser axotomies were performed in animals carrying the *vdEx662* transgene in 0.05% tetramisole in M9 and 4% agar pads. Bidirectional confocal imaging for pre- and postaxotomy samples was conducted for both WT and mutant animals on the same agar pad using 11% laser power for 488 nm and 0.6% for 561 nm. Animals were imaged before axotomy and at 15 min, 1 h, 4 h, 8 h, and 24 h after injury as stated in Neumann et al. (3), using the Airyscan Multiplex (MPLX)-Super Resolution (SR) 8Y mode, and representative images were taken using the Airyscan MPLX-SR-4Y mode on the Zeiss LSM 980 compound microscope (Zeiss Group, Oberkochen, Germany) and Zen 2 software.

### Expression of *Peff-1::GFP*

Confocal imaging was performed to track transcriptional activity of EFF-1 in WT and *dyn-1* cell bodies of the PLM neuron. The strain QH4313 *(zzIs16 [pJE3 (Peff-1::GFP) + pRF4 (rol-6(su1006))]; vdEx263 (5 ng/uL Pmec-4::mCherry + 30 ng/uL Podr-1::dsRed + 65 ng/uL pSM))* was crossed into the *dyn-1(ky51)* mutant background. To allow the PLM neurons to be visualized, RNAi was used to knockdown GFP in the muscles and intestine. This was achieved through a feeding method as previously described (42). Briefly, L4 stage animals were placed on IPTG-containing plates seeded with *Escherichia coli* [(HT115(DE3)] expressing dsRNA of GFP. As the *C. elegans* nervous system is resistant to RNAi knockdown (43, 44), levels of GFP in the PLM neuron are not affected by this approach.

To examine EFF-1 expression levels in the cell body, animals were first incubated at 25°C for at least 2 h prior to imaging. Animals were immobilized with 0.05% tetramisole in M9 on 4% agar pads. Bidirectional confocal imaging for pre- and 1 h postaxotomy samples was conducted for both WT and mutant animals on the same agar pad using 11% laser power for 488 nm and 7.5% for 561 nm. Animals were imaged using the Airyscan Multiplex (MPLX)-Super Resolution (SR) 8Y mode on the Zeiss LSM 980 compound microscope (Zeiss Group, Oberkochen, Germany) and Zen 2 software.

To measure transcriptional activity, the cell body was traced and the mean fluorescence, area, and integrated density were recorded using ImageJ software (National Institutes of Health, Bethesda, MD, USA). We then consistently traced an area of 58.169 µm^2^ (length × width = 8.52 µm × 8.52 µm) outside the worm body in all images to measure the background signal. Due to the complexity of the experiment, we examined at least nine animals per genotype to measure the CTCF. The CTCF per sample was calculated using the following formula: CTCF = integrated density − (area of the traced cell body × mean fluorescence of the background signal).

### Statistical analyses

Statistical analyses were performed using GraphPad Prism 9.0 (CA, USA). Standard error of proportion and Fischer's exact tests were used to calculate statistical values for axonal fusion events where *P < 0.05, **P < 0.005, and ***P < 0.001. To compare regrowth length defects, *Pmec-4::EFF-1::GFP* and *Peff-1::GFP* intensities, standard unpaired *t* tests were used where *P < 0.05, **P < 0.005, and ***P < 0.001.

## Supplementary Material

pgad114_Supplementary_Data

## Data Availability

All relevant data are within the paper and its supplementary files.
