# Supplementary material for: The dynamin GTPase mediates regenerative axonal fusion in Caenorhabditis elegans by regulating fusogen levels
Source: PNAS Nexus. 2023 May 9;2(5):pgad114. doi: 10.1093/pnasnexus/pgad114 (PMC10167995; doi:10.1093/pnasnexus/pgad114)
Supplement: pgad114_Supplementary_Data [file pgad114_supplementary_data.pdf]

Figure S1

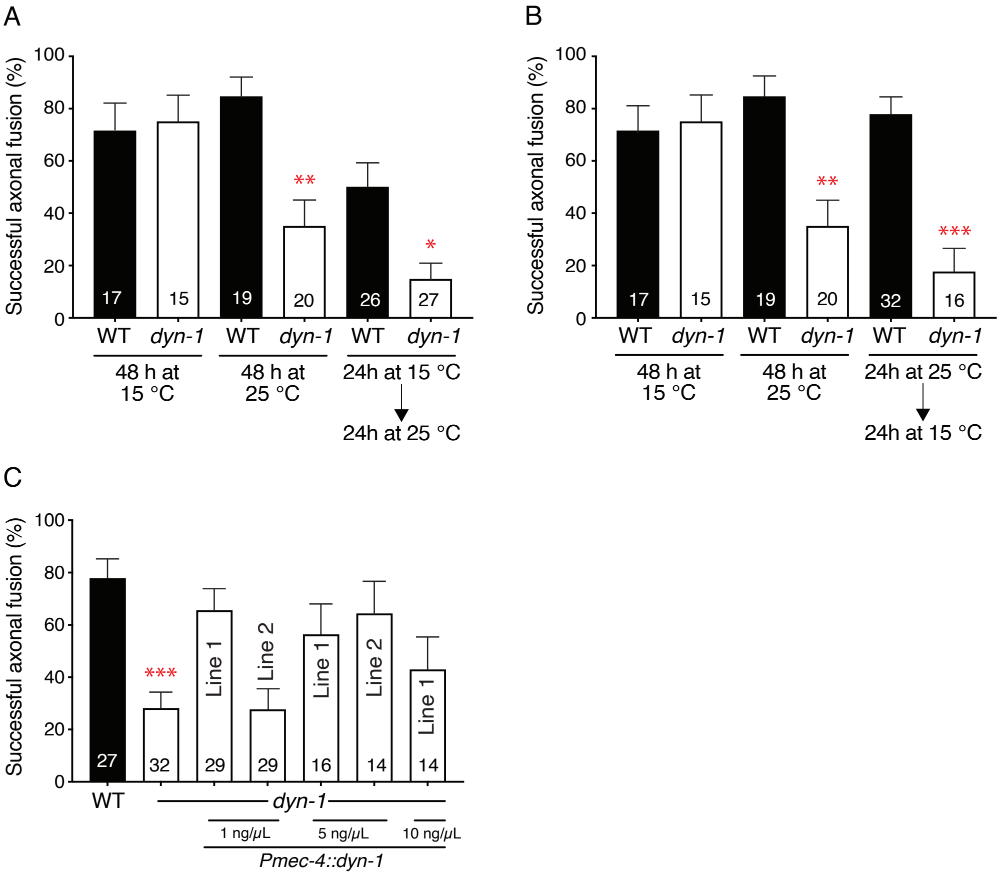

Figure S1: DYN-1 is required for several hours after injury to promote axonal repair. A, B) Quantification of axonal fusion levels in animals grown and incubated at 15°C post-injury (left most bars), in animals grown at 15°C and incubated at 25°C after injury (middle bars), or in animals grown at 15°C and incubated according to the corresponding experimental design, post-injury (right bars). Error bars represent standard error of proportion; P-values calculated using Fisher's exact tests; $*p<0.05$, $**p<0.005$, $***p<0.001$, $****p<0.0001$. Numbers within individual bars indicate the number of reconnection events per genotype, where n ≥15. **C)** Axonal fusion events in *dyn-1(ky51)* lines expressing extrachromosomal rescue arrays. Varying concentrations of *Pmec-4::dyn-1* plasmids were injected into *dyn-1(ky51)* animals. Error bars represent standard error of proportion; P-values calculated using Fischer's exact tests; $p***<0.001$. Numbers within individual bars indicate the number of reconnection events per genotype, where n ≥14 per genotype.

Figure S2

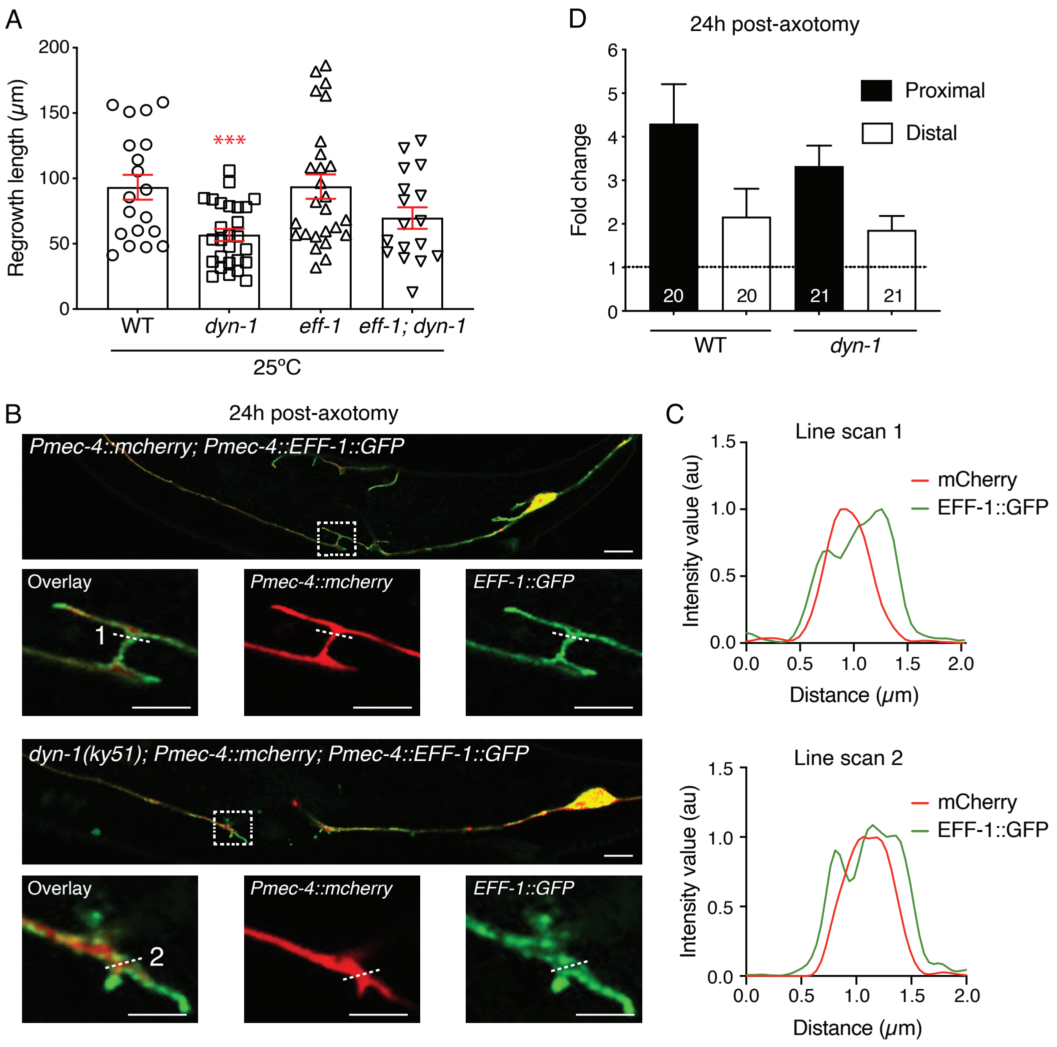

Figure S2: EFF-1 expression levels are transiently altered after injury. A) Graph representing regrowth lengths of single *dyn-1(ky51)* or *eff-1(ok1021)* mutants and double *eff-1(ok1021); dyn-1(ky51)* mutants at 25ºC. Loss of EFF-1 does not affect regrowth length in single or double mutants, suggesting that EFF-1 and DYN-1 do not regulate regrowth length via the same genetic pathway. Regrowth length statistics calculated using unpaired student t-tests; ***p<0.001, bars represent mean ± SE. **B)** Analysis of Pmec-4::EFF-1::GFP distribution in WT and *dyn-1(ky51)* animals post-axotomy. Animals were incubated for a minimum of 2 hours at 25°C before pre-axotomy images were taken and transferred to 25°C for 24 h post-axotomy. Bars represent 5 μm. Dotted white boxes (above) represent the area of physical contact between the two cut segments and dotted white lines (below) represent cross-sectional area to assess EFF-1::GFP distribution in comparison to the mCherry reference channel. **C)** Corresponding normalized line scans of EFF-1::GFP and mCherry in WT and *dyn-1(ky51)* animals from (B). EFF-1::GFP is distributed on either side of the reference channel in WT and *dyn-1(ky51)* animals 24 hours after axotomy, indicating a membrane localization. **D)** EFF-1::GFP fold change relative to pre-axotomy images in WT and *dyn-1(ky51)* animals measured approximately 14-15 μm from the cut site after 24 hours of injury, n ≥ 20 animals.

Figure S3

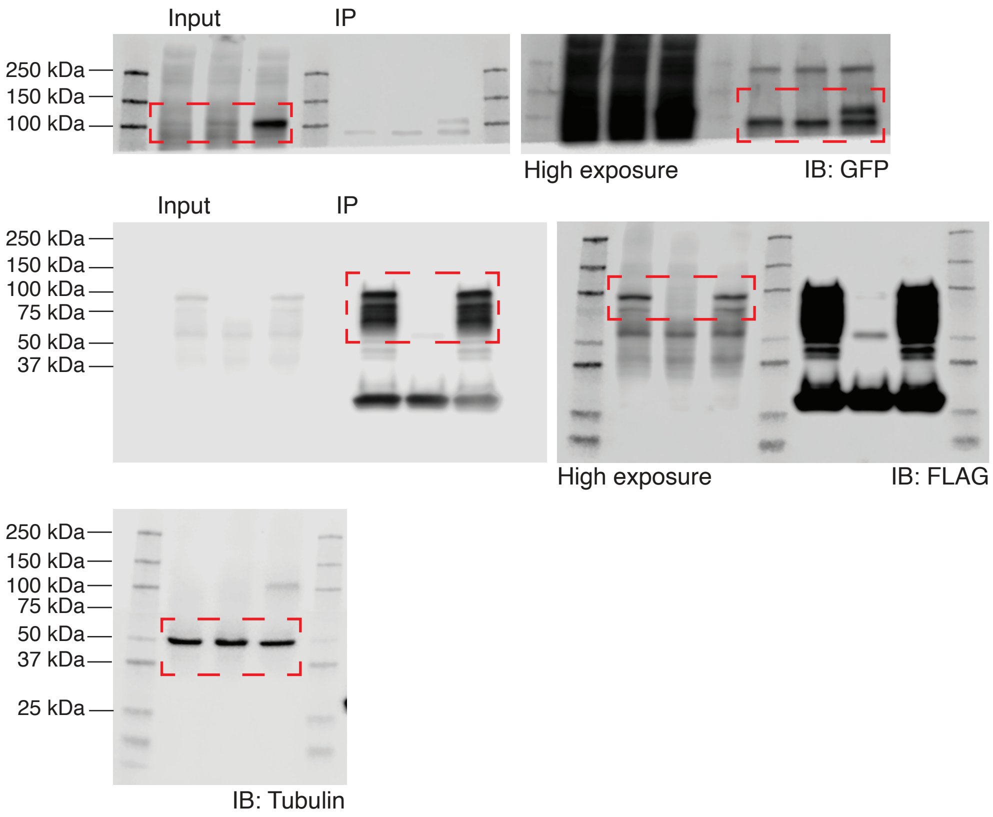

Figure S3: Uncropped western blot images. Related to Fig. 3C. DYN-1A-3XFLAG and EFF-1A-eGFP were expressed in HEK293T cells. Immunoprecipitation of DYN1A-3xFLAG using anti-FLAG antibodies.

Supplementary Table 1. The *C. elegans* strains used in this study.

Strain	Genotype	Origin
**QH3135**	*zdIs5(Pmec-4::GFP)*	Hilliard lab
**QH3192**	*dyn-1(ky51); zdIs5(Pmec-4::GFP)*	Hilliard lab
**QH4748**	*eff-1(ok1021); vdEx662(Pmec-4::EFF-1::GFP; Podr-1::DsRed; Pmec-4::mCherry)*	Hilliard lab
**QH4313**	*zzIs16[pJE3 (Peff-1::GFP) + pRF4 (rol-6(su1006))]; vdEx263 (5 ng/uL Pmec-4::mCherry + 30 ng/uL Podr-1::dsRed + 65 ng/uL pSM)*	Hilliard lab
**PHX2717**	*sybIs2534(Pmec-17::3xFLAG::dyn-1a cDNA::rab-3 3'UTR); zdIs5(Pmec-4::GFP)*	SunyBiotech
**PHX2689**	*sybIs2535(Pmec-17::3xFLAG::dyn-1b cDNA::rab-3 3'UTR); zdIs5(Pmec-4::GFP)*	SunyBiotech
**BXN342**	*eff-1(ok1021); zdIs5(Pmec-4::GFP)*	Neumann lab
**BXN344**	*eff-1(ok1021); dyn-1(ky51); zdIs5(Pmec-4::GFP)*	Neumann lab
**BXN762**	*dyn-1(ky51); eff-1(ok1021); vdex662(Pmec-4::EFF-1::GFP; Pmec-4::mCherry; Podr-1::dsRed)*	Neumann lab
**BXN797**	*dyn-1(ky51); sybIs2534(Pmec-17::3xFLAG::dyn-1a cDNA::rab-3 3'UTR); zdIs5(Pmec-4::GFP)*	Neumann lab
**BXN818**	*dyn-1(ky51); sybIs2535(Pmec-17::3xFLAG::dyn-1b cDNA::rab-3 3'UTR); zdIs5(Pmec-4::GFP)*	Neumann lab
**BXN863**	*dyn-1(ky51); zdIs5(Pmec-4::GFP); cjnEx158 (1 ng/uL Pdyn-1::dyn-1::wrmScarlet)*	Neumann lab
**BXN917**	*dyn-1(ky51); zdIs5(Pmec-4::GFP); zzIs16[pJE3 (Peff-1::GFP) + pRF4 (rol-6(su1006))]; vdEx263 (5 ng/uL Pmec-4::mCherry + 30 ng/uL Podr-1::dsRed + 65 ng/uL pSM)*	Neumann lab
